# Comparative Study on Mechanical Performance and Toughness of High-Performance Self-Compacting Concrete with Polypropylene and Basalt Fibres

**DOI:** 10.3390/ma18163833

**Published:** 2025-08-15

**Authors:** Piotr Smarzewski, Anna Jancy

**Affiliations:** Faculty of Civil Engineering and Geodesy, Military University of Technology, 2 Gen. Sylwestra Kaliskiego, 00908 Warsaw, Poland

**Keywords:** self-compacting concrete (SCC), basalt fibre (BF), polypropylene fibre (PP), high-performance concrete (HPC), mechanical properties, toughness, fracture behavior, fibre reinforced concrete (FRC), ground granulated blast furnace slag (GGBFS), rheological properties

## Abstract

This study investigates the flexural performance, tensile splitting strength, and fracture behaviour of self-compacting concrete (SCC) reinforced with polypropylene (PP) and basalt (BF) fibres. A total of eleven SCC mixtures with varying fibre types and volume fractions (0.025–0.25%) were tested at 7 and 28 days. In this study, the term high-performance concrete (HPC) refers to SCC mixtures with a 28-day compressive strength exceeding 60 MPa, as commonly accepted in European standards and literature. The control SCC achieved 68.2 MPa at 28 days. While fibre addition enhanced the tensile and flexural properties, it reduced workability, demonstrating the trade-off between mechanical performance and flowability in high-performance SCC. The experimental results demonstrate that both fibre types improve the tensile behaviour of SCC, with distinct performance patterns. PP fibres, owing to their flexibility and crack-bridging capability, were particularly effective at early ages, enhancing the splitting tensile strength by up to 45% and flexural toughness by over 300% at an optimal dosage of 0.125%. In contrast, BF fibres significantly increased the 28-day toughness (up to 15.7 J) and post-cracking resistance due to their superior stiffness and bonding with the matrix. However, high fibre contents adversely affected workability, particularly in BF-reinforced mixes. The findings highlight a dosage-sensitive behaviour, with optimum performance observed at 0.05–0.125% for PP and 0.125–0.25% for BF. While PP fibres improve crack distribution and early-age ductility, BF fibres offer higher stiffness and energy absorption in post-peak regimes. Statistical analysis (ANOVA and Tukey’s test) confirmed significant differences in the mechanical performance among fibre-reinforced mixes. The study provides insights into selecting appropriate fibre types and dosages for SCC structural applications. Further research on hybrid fibre systems and long-term durability is recommended. The results contribute to sustainable concrete design by promoting enhanced performance with low-volume, non-metallic fibres.

## 1. Introduction

Self-compacting concrete (SCC) exhibits distinct properties in both fresh and hardened states compared to conventional concrete, primarily due to its ability to flow and compact under its own weight without vibration. This property allows concrete to fill complex formwork shapes and densely reinforce structures effectively [1]. A key aspect of SCC is the use of additional cementitious materials such as ground granulated blast furnace slag (GGBS), which reduces cement consumption, improves durability, lowers permeability, and improves chemical stability by reacting with soluble calcium hydroxide [2,3]. Recent studies have also explored alkali-activated binders that incorporate industrial by-products, such as thermally activated red mud, to further enhance mechanical and durability performance [4]. However, the dense microstructure of high-performance concrete (HPC), while beneficial for compressive strength and permeability, typically results in increased brittleness and a reduced resistance to fire [5]. According to key textbooks [6,7], concrete with a compressive strength greater than 55–60 MPa is considered high-performance. Consequently, the mixtures in this study meet the HPC criteria.

In order to address these issues, fibre reinforcement has been widely adopted in HPC to improve its tensile strength, toughness, and crack resistance [8,9]. Among various types of fibres, polypropylene fibres (PP) and basalt fibres (BF) have attracted significant attention due to their beneficial properties. Polypropylene fibres are known for their low density, excellent chemical stability, hydrophobicity, and effectiveness in controlling plastic shrinkage cracks, although they typically offer limited improvements in mechanical strength due to their relatively low tensile strength and weak bonding with the cementitious matrix [10,11,12,13,14]. However, PP fibres improve the toughness, flexural strength, and splitting tensile strength of concrete, especially in moderate volumes of fibre, by controlling crack development [15,16,17,18,19].

In contrast, basalt fibres exhibit high tensile strength, excellent thermal resistance, and strong durability in alkaline environments, positioning them as an environmentally friendly and cost-effective reinforcement alternative to conventional steel fibres [20,21,22,23]. They significantly improve the flexural strength, fracture toughness, and energy absorption of cement-based composites, despite adversely affecting the workability of fresh concrete compared to other fibres [21,24,25,26]. Previous studies have indicated that optimal the mechanical performance for BF-reinforced SCC is achieved within a fibre volume range of approximately 0.25 to 0.5%, although research specifically addressing SCC mixtures remains limited [21,26].

To date, extensive investigations of SCC reinforced separately with PP or BF fibres have been carried out. However, direct comparative studies with identical concrete matrices and consistent fibre volumes remain scarce. Smarzewski [27] previously investigated high-performance SCC incorporating polypropylene fibres and high-volume GGBS, demonstrating improvements in toughness, flexural, and tensile properties. In parallel, Smarzewski [28] studied basalt fibre-reinforced SCC, observing beneficial effects on fracture toughness, flexural strength, and mechanical performance. However, a critical review of the available literature reveals several important shortcomings. First, most existing studies have independently evaluated either polypropylene or basalt fibres in SCC, with only a limited number attempting direct comparisons using the same matrix composition and testing conditions. Comparative investigations often suffer from inconsistencies in the design of concrete mixes, the dose of fibres, and the test protocols, making it difficult to draw reliable conclusions about the specific effects of each fibre type. Moreover, while the majority of previous research has focused on normal vibrated concrete or conventional HPC, the unique behaviour of fibre-reinforced SCC, notably its sensitivity to changes in fibre-induced workability, has received less attention. Additionally, many studies lack detailed statistical analyses that objectively quantify the impact of fibre type and content, and rarely address the balance between mechanical performance and workability, especially at higher fibre doses. Therefore, a systematic, side-by-side evaluation of PP and BF fibres in SCC, using a unified experimental design and robust statistical methods, remains a clear gap in the field. The present study aims to address these issues directly.

Given the existing literature gap, the present study aims to directly compare the mechanical performance, toughness, and fresh properties of high-performance SCC reinforced with polypropylene and basalt fibres with identical fibre contents and concrete matrix compositions. This comparative assessment aims to quantify and clarify the impact of each type of fibre on the workability and mechanical characteristics of SCC. In addition, the paper seeks to identify the optimal fibre content that maximises the mechanical and durability performance, facilitating broader adoption in structural applications.

## 2. Materials and Methods

### 2.1. Materials

The raw materials used to prepare all SCC mixtures included ordinary Portland cement (CEM I 42.5R), ground granulated blast furnace slag (GGBS), quartz sand (FA), gravel aggregate (CA), tap water (W), and high-range water-reducing admixtures (HRWAs). The same SCC matrix was used for all mixtures, differing only in the type and volume fraction of the fibres (PP or BF). The chemical compositions of cement and GGBS are presented in Table 1.

**Figure 1:**
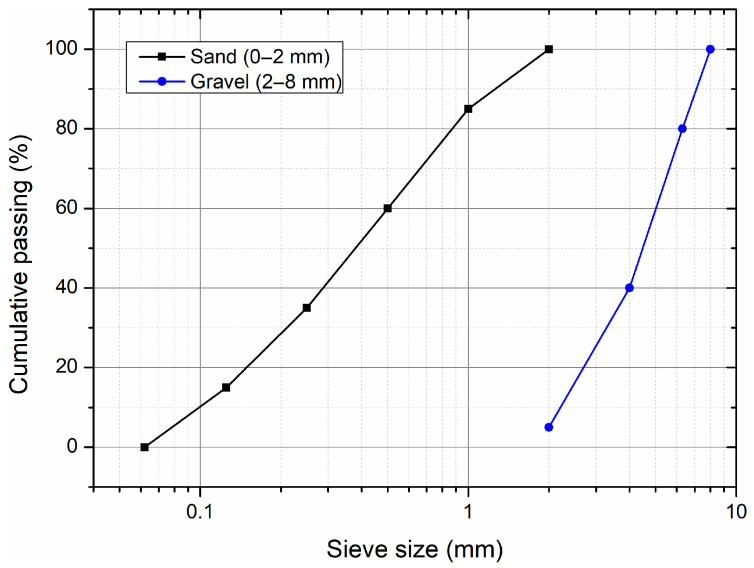
Particle size distribution curves for sand (0–2 mm) and natural gravel (2–8 mm) used in the experimental program.

The particle size distribution (PSD) of both fine and coarse aggregates was determined according to PN-EN 933-1 [29]. The PSD curves are shown in Figure 1. The coarse aggregate was natural, rounded gravel with a size fraction of 2 to 8 mm, and the fine aggregate (plastering sand) was predominantly subangular. The water absorption of gravel and sand was 1.5% and 1.2%, respectively.

The HRWAs were based on lignosulfonates and polycarboxylic ethers (ISOLA BV, ISOFLECX 833 and ISOFLOW 755) by CEMEX (Salzkotten, Germany) , dosed at 3% of the binder mass. The mix design was optimised to maintain a target slump flow of 720 ± 10 mm (class SF2).

The fibre properties are summarised in Table 2.

### 2.2. Mix Proportions

All SCC mixtures shared the same matrix (cementitious binder 650 kg/m^3^, W/B ratio 0.32) and differed only in the type and volume fraction of the fibre. The fibre content was varied between 0.025% and 0.25% by volume. Lower dosages were not considered because they would have little to no impact on crack resistance, while higher dosages were avoided due to their detrimental effect on flowability, as evidenced by the failure of slump flow and T_500_ criteria in preliminary trials. This limitation is particularly significant for SCC with a maximum aggregate size of 8 mm, as an excessive fibre content can lead to the severe loss of workability and an increased risk of segregation. The mixture design is shown in Table 3.

### 2.3. Mixing Procedure

The sequence of mixing was as follows: (1) coarse and fine aggregates were mixed for 2 min, (2) cement and GGBS were added for 2 min, (3) water and HRWA were mixed and added for 4 min, and (4) fibres were introduced and mixed for another 4 min. The fibres were introduced manually and evenly during the final stage of mixing, and gradually spread over the surface of the fresh concrete. After addition, the mixing continued for an additional 4 min to promote uniform dispersion and minimise agglomeration. All mixtures were prepared in a laboratory pan mixer with a total mixing time of approximately 12 min. Fresh concrete was tested immediately.

### 2.4. Testing Procedure

The properties of fresh concrete were assessed through slump flow (Ø and T_500_) and L-box (passing ability, PA), according to the European Guidelines for SCC [1].

The hardened properties were tested at 7 and 28 days. The compressive strength was determined on 100 mm cubes per PN-EN 12390-3 [30] with a loading rate of 0.5 MPa/s. The splitting tensile strength was measured according to PN-EN 12390-6 [31], using a rate of 0.05 MPa/s. The flexural strength was tested under a three-point bending configuration according to PN-EN 12390-5 [32], using 100 × 100 × 500 mm prisms and a displacement-controlled rate of 0.05 mm/min.

The post-cracking behaviour and flexural toughness were evaluated in accordance with ASTM C1609 [33]. From the load–deflection curves, parameters such as toughness (area under the curve) were determined for each specimen.

For each mix composition, three samples were prepared and tested for each experimental procedure (compressive strength, splitting tensile strength, flexural strength, and toughness) at 7 and 28 days of curing. The results presented are the mean values of the three measurements.

## 3. Results

### 3.1. Properties of Fresh Mixes

The fresh properties of self-compacting concretes (SCC) incorporating polypropylene (PP) and basalt (BF) fibres are presented in Table 4. These parameters include the diameter of the slump flow, the T_500_ time, and the blocking ratio of the L-box, which reflect the filling ability, the viscosity, and the passing ability, respectively. The control mix (SCC-REF) achieved the highest flowability, with a slump flow of 722.5 mm and a passing ability of 0.94, confirming excellent rheological behaviour.

The inclusion of PP fibres reduced the flowability of SCC, with the diameters of the slump flow decreasing as the fibre content increased. Mixtures with 0.025–0.05% vol. of PP fibres maintained the SF2 classification (660–750 mm), suitable for conventional applications like walls and columns. However, mixes with 0.075–0.125% vol. PP showed reduced slump flow (580.5–570 mm), falling into the SF1 class (550–650 mm), typically used in more restricted or congested structural elements like tunnel linings or deep foundations. The most fibre-rich mix (SCC-PP-0.25) dropped below the minimum flow requirements (485 mm), confirming that an excessive PP fibre content severely deteriorates flowability. This trend confirms observations by previous researchers who reported an increasing viscous resistance with greater PP fibre volumes [8,9,17]. The T500 values increased from 4.1 to 5.0 s, indicating heightened mixture viscosity. According to the European guidelines [1], these mixtures fall under VS2 class (T_500_ between 2–5 s). The L-box blocking ratios ranged from 0.90 to 0.82 for moderate PP contents, and dropped to 0.63 for SCC-PP-0.25, indicating that a higher fibre content significantly reduces passing ability.

Similarly to PP, basalt fibres also adversely influenced the fresh properties of SCC. The slump flow progressively decreased from 680 mm (0.025% BF) to 478.5 mm (0.25% BF), with mixes above 0.125% BF falling below SF1 limits. SCC-BF-0.025 met the SF2 classification, while SCC-BF-0.05 to SCC-BF-0.125 were within SF1, demonstrating that BF addition must be carefully controlled. The lowest flow diameter for SCC-BF-0.25 indicates severe restriction to flow, consistent with earlier findings [24,27]. The T_500_ times for BF mixes ranged between 4.4 and 5.9 s, suggesting a transition from VS2 to borderline VS3 (high viscosity). As with PP fibres, the increase in T_500_ confirms that the mixtures become more viscous with a higher fibre content. The L-box passing ability decreased with increasing BF content, dropping from 0.89 to 0.61, again indicating poor deformability and flow through congested spaces.

When comparing the impact of polypropylene (PP) and basalt fibres (BF) on the rheological properties of SCC mixtures, distinct differences are observed despite identical matrix compositions and fibre volume fractions. At comparable doses, PP fibres caused a moderate reduction in the diameter of the slump flow and the passing ability. Even at 0.125% vol., the slump flow remained within the SF1 class, and the L-box ratio stayed above the threshold of 0.80, indicating that PP fibres, due to their low stiffness and hydrophobic surface, have a less disruptive effect on workability. Their crimped geometry and low density contribute to lower resistance to flow and improved dispersion in the matrix. In contrast, basalt fibres significantly deteriorated the workability of SCC, especially at higher volume fractions. Even at 0.075% and 0.125% vol., the mixtures showed reduced slump flow and lower blocking ratios compared to their PP counterparts. The stiff, high-density, and smooth-surfaced basalt fibres tend to cluster and increase internal friction during flow, resulting in higher viscosity and reduced deformability. The SCC-BF-0.25 mixture completely failed to meet SCC workability standards, confirming the more adverse effect of basalt fibre inclusion on rheology.

In summary, PP fibres allow greater flexibility in the formulation of SCC and maintain acceptable rheological behavior up to 0.125% vol., whereas BF-reinforced SCCs show higher viscosity and decreased flowability even at lower fibre contents, requiring careful mix design adjustments to preserve SCC characteristics.

### 3.2. Compressive Strength

**Figure 2:**
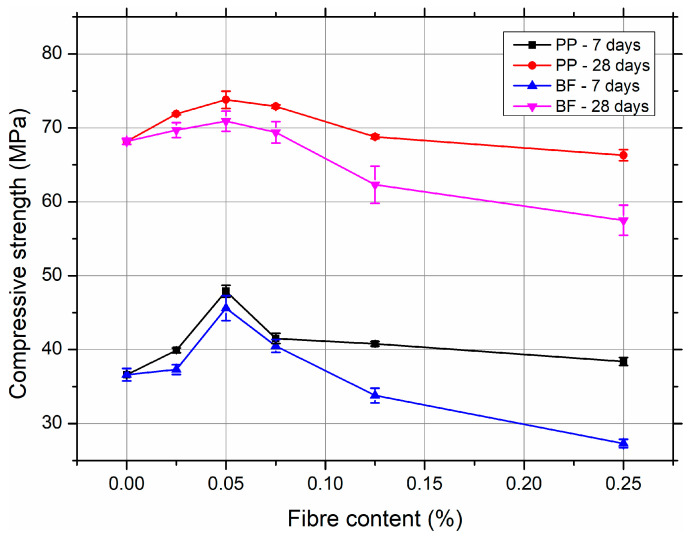
Effect of fibre type and volume content on the compressive strength of SCC after 7 and 28 days of curing. Detailed numerical results, including standard deviations and coefficients of variation, are provided in the Supplementary Material (Table S1).

Figure 2 illustrates the development of compressive strength in self-compacting concrete (SCC) reinforced with polypropylene (PP) and basalt fibres (BF) in various volume fractions, evaluated after 7 and 28 days of curing.

The inclusion of PP fibres at low doses (up to 0.05% by volume) resulted in a marked improvement in compressive strength compared to the reference SCC without fibres. The optimum strength was observed at 0.05% PP fibre content, reaching 47.9 MPa at 7 days and 73.8 MPa at 28 days, corresponding to increases of approximately 31% and 8%, respectively, compared to the control mix. These improvements can be attributed to the fibre’s ability to reduce microcracking and enhance the internal stress distribution during early hydration. However, at higher fibre dosages (≥0.075%), a gradual reduction in strength was observed, likely due to reduced workability, air entrapment, and heterogeneity in fibre distribution, which adversely affect matrix integrity.

Similarly, SCC reinforced with basalt fibres exhibited an initial increase in compressive strength with an optimum volume content of 0.05% (45.6 MPa at 7 days and 70.9 MPa at 28 days). Nevertheless, a significant decline in compressive strength occurred with an increasing fibre content beyond this point. At the highest dosage of 0.25%, the compressive strength dropped by approximately 25% at 7 days and ~16% at 28 days compared to the reference mix. This deterioration is likely due to the pronounced stiffening and reduced fluidity of the fresh mix induced by the rigid and dense basalt fibres, leading to poor compaction and an increased void ratio in hardened concrete.

In comparison, PP fibres maintained better compressive strength retention at higher contents than basalt fibres, suggesting a more favourable balance between fibre dispersion and rheological stability. Both types of fibres demonstrated a clear dosage-sensitive behaviour, with strength enhancement occurring only within an optimal range. These findings highlight the need to control the fibre content to avoid detrimental effects on workability and mechanical performance.

### 3.3. Splitting Tensile Strength

**Figure 3:**
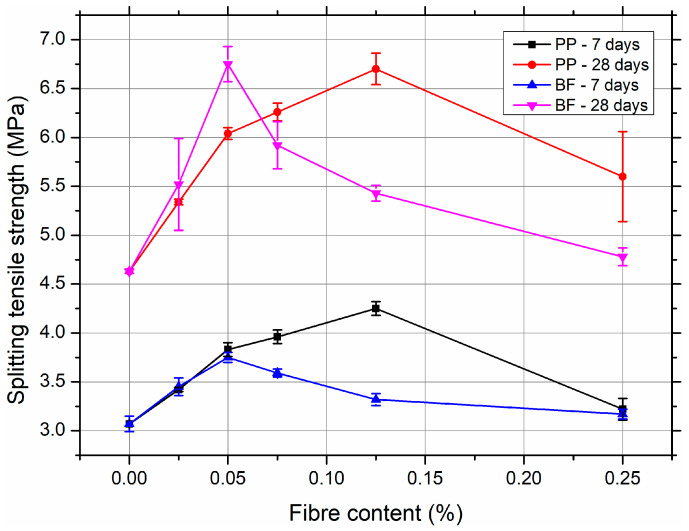
Splitting tensile strength of SCC mixtures reinforced with PP and BF after 7 and 28 days of curing. Detailed numerical results, including standard deviations and coefficients of variation, are provided in the Supplementary Material (Table S2).

The evolution of the splitting tensile strength for the SCC mixtures reinforced with polypropylene (PP) and basalt fibres (BF) is illustrated in Figure 3 for 7 and 28 days of curing.

The incorporation of PP fibres significantly enhanced the tensile performance of SCC. At an early age (7 days), a consistent increase in strength was observed with increasing fibre content, reaching a maximum of 4.25 MPa at 0.125% PP content—an increase of 38% relative to the reference SCC (3.07 MPa). At 28 days, the peak splitting tensile strength was 6.70 MPa at the same fibre dosage, representing a 45% improvement over the control mix. This can be attributed to the ability of PP fibres to effectively bridge microcracks, delay crack propagation, and improve stress redistribution under tensile loading. However, at the highest content (0.25%), a noticeable reduction was observed, likely due to fibre clustering and reduced matrix integrity, as evidenced by the increased coefficient of variation (CV = 8.32%).

For SCCs reinforced with basalt fibres, the tensile strength also improved as the fibre content increased to 0.05%, peaking at 3.75 MPa (7 days) and 6.75 MPa (28 days), representing increases of 22% and 46%, respectively, over the reference. Beyond this dosage, however, a gradual decline in tensile performance was observed. This can be attributed to the stiff and hydrophilic nature of basalt fibres, which can adversely affect fibre dispersion and matrix cohesion at higher dosages. Nevertheless, BF-reinforced SCCs showed slightly superior tensile performance compared to PP-reinforced mixes at 28 days for equivalent fibre dosages (0.05%), but exhibited greater variability (higher SD and CV), indicating less stable composite behaviour at elevated contents.

In summary, both types of fibre contributed positively to the splitting tensile strength of SCC at moderate doses, with an optimal range identified around 0.05 to 0.125% volume. The beneficial effect diminished at higher fibre contents due to compromised workability and fibre distribution.

### 3.4. Flexural Strength

**Figure 4:**
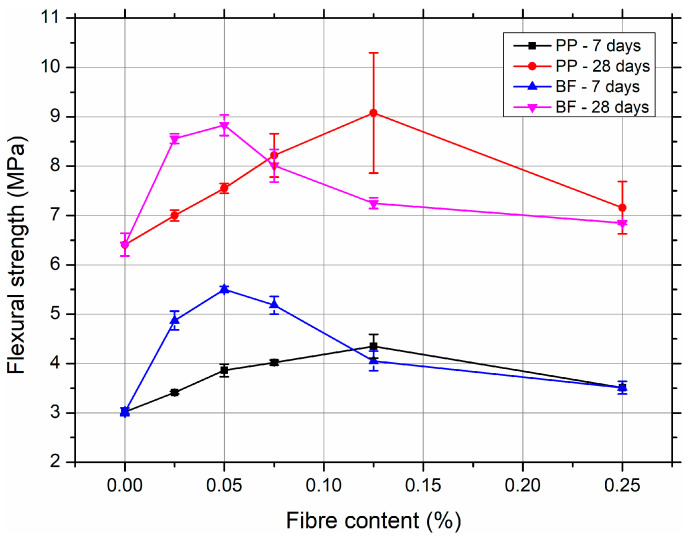
Flexural strength of SCC mixtures reinforced with PP and BF after 7 and 28 days of curing. Detailed numerical results, including standard deviations and coefficients of variation, are provided in the Supplementary Material (Table S3).

The results of the flexural strength measurements for the SCC mixtures reinforced with PP and BF after 7 and 28 days of curing are presented in Figure 4.

At an early curing age (7 days), the addition of PP fibres gradually enhanced the flexural strength up to a 0.125% fibre content, where the peak value of 4.35 MPa was recorded, an increase of 44% compared to the control mix (3.02 MPa). A similar trend was observed at 28 days, with the strength improving from 6.41 MPa (SCC-REF) to 9.08 MPa at a 0.125% fibre content. However, a further increase to 0.25% PP fibre led to a reduction in flexural strength, likely due to increased heterogeneity and impaired matrix cohesion. Additionally, the higher standard deviation (SD = 1.22 MPa) and coefficient of variation (CV = 13.44%) at 0.125% indicate greater variability in the composite performance at elevated fibre volume fractions. The relatively high standard deviation observed at the 0.125% PP fibre dosage may be attributed to the increased likelihood of fibre clustering or agglomeration during mixing, which can locally alter the homogeneity of the matrix. This effect is especially pronounced in SCC, where workability and fibre dispersion are sensitive to even moderate increases in fibre content.

The SCC mixes reinforced with basalt fibres exhibited overall higher flexural strengths compared to those with PP fibres at comparable dosages. The highest value was achieved at 0.05% BF content, reaching 5.50 MPa (7 days) and 8.83 MPa (28 days), marking an increase of 82% and 38%, respectively, over the reference mix. Nonetheless, beyond this optimal dosage, a gradual decline in flexural strength was observed. This may be attributed to the reduced workability and suboptimal fibre dispersion at higher BF contents, despite the high intrinsic stiffness and strength of the fibres. Interestingly, BF-reinforced mixes showed lower CV values at 28 days compared to PP mixes at higher dosages, indicating more consistent behaviour, particularly at 0.25% BF (CV = 0.38%).

In general, both types of fibre positively influenced flexural strength, with optimal dosages around 0.05 to 0.125%. Basalt fibres generally provided superior strength improvements but demonstrated slightly less tolerance to overdosing in terms of strength retention.

### 3.5. Load–Displacement Behaviour and Flexural Toughness

**Figure 5:**
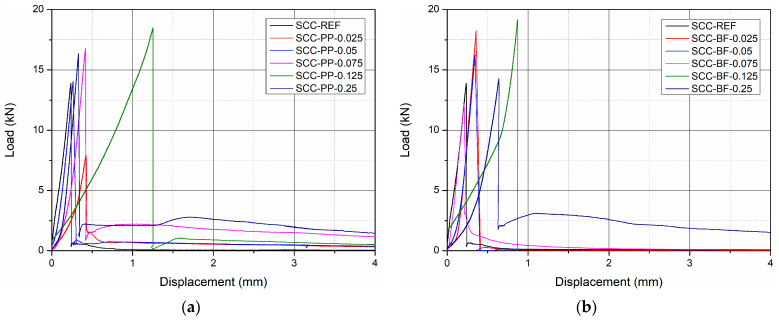
Typical load–displacement behaviour of SCC mixtures reinforced with (**a**) PP and (**b**) BF after 28 days of curing.

Figure 5 presents the typical load–displacement behaviour of self-compacting concrete (SCC) mixtures reinforced with polypropylene (PP) fibres (a) and basalt fibres (BF) (b) after 28 days of curing. The curves illustrate the differences in the post-cracking behaviour and energy absorption capacity (flexural toughness) depending on the type and content of the fibre.

In general, all fibre-reinforced mixtures showed improved post-cracking performance compared to the plain reference mix (SCC-REF), which exhibited a typical brittle failure with a sudden drop in load immediately after the peak. In contrast, fibre-reinforced composites exhibited various degrees of stress redistribution and residual load-bearing capacity after cracking. For concrete reinforced with PP (Figure 5a), increasing the volume fraction of the fibre resulted in a more ductile response, with higher deflections and an extended load-carrying capacity beyond the peak. In particular, the SCC-PP-0.075 and SCC-PP-0.125 mixtures demonstrated a pronounced plateau and steady load-carrying performance, indicating effective crack-bridging and energy dissipation mechanisms provided by the PP fibres. The SCC-PP-0.25 mix showed the most stable post-peak response, albeit with slightly reduced peak load, suggesting improved toughness but possible compromises in early-age strength or workability. For concrete reinforced with BF (Figure 5b), the behaviour was markedly different. Although peak loads were higher for mixtures such as SCC-BF-0.05 and SCC-BF-0.075, the post-cracking behaviour was generally more abrupt and less ductile compared to PP-reinforced SCC. The load dropped sharply after the peak in most BF mixtures, with a limited capacity for stress transfer after matrix cracking. An exception was SCC-BF-0.25, which showed extended deformation at lower residual loads, suggesting partial fibre extraction or fracture mechanisms.

These findings confirm the distinct roles of fibre type: PP fibres enhance ductility and toughness through bridging and plastic deformation, while BF fibres tend to increase strength but offer limited post-crack energy absorption due to their brittle nature and lower elongation at break.

**Figure 6:**
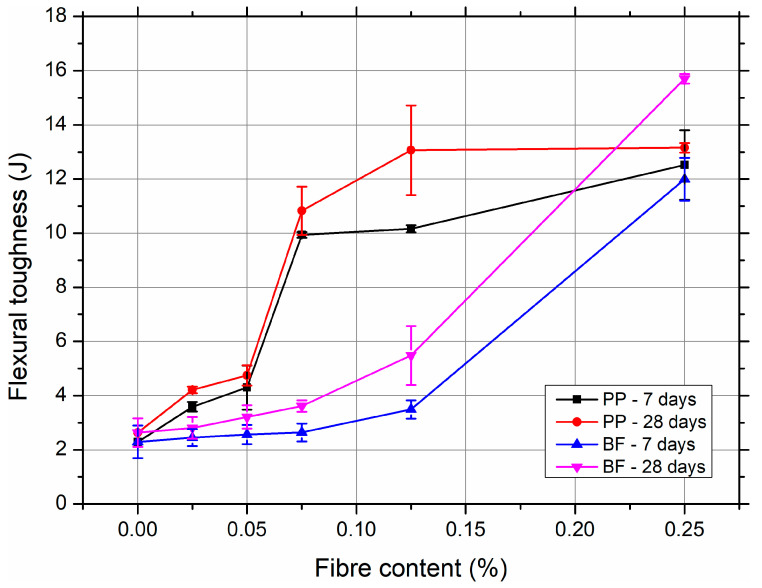
Flexural toughness of SCC mixtures reinforced with PP and BF after 7 and 28 days of curing. Detailed numerical results, including standard deviations and coefficients of variation, are provided in the Supplementary Material (Table S4).

The qualitative observations of the load–displacement curves (Figure 5) are further supported by the quantitative results of the flexural toughness summarised in Figure 6.

As expected, the incorporation of fibres significantly increased the energy absorption capacity of SCC mixtures compared to the plain reference (SCC-REF), which exhibited toughness values of only 2.29 J and 2.63 J at 7 and 28 days, respectively. The flexural toughness was calculated as the area under the load–deflection curve, expressed in joules (J), assuming constant specimen geometry.

For polypropylene fibre-reinforced concretes, a pronounced increase in flexural toughness was observed with increasing fibre volume. At just 0.075% volume content, toughness jumped to 9.94 J at 7 days and 10.83 J at 28 days, reflecting a nearly fourfold increase over the reference. The improvement continued up to a 0.25% fibre content, where the toughness reached a peak of 13.15 J at 28 days. This trend confirms the high efficiency of PP fibres in enhancing the post-cracking energy dissipation due to their ability to bridge cracks and undergo plastic deformation. The relatively low coefficient of variation (CV) for PP mixtures, particularly at higher dosages (e.g., 1.07% for SCC-PP-0.075), indicates stable and repeatable toughening behaviour.

In contrast, SCC mixtures reinforced with basalt fibres (BF) exhibited a more modest improvement in toughness at lower dosages. At a 0.05% and 0.075% BF content, the flexural toughness increased only slightly compared to the reference, suggesting limited fibre engagement in the post-cracking regime. However, at a 0.25% content, a substantial increase was recorded, up to 15.70 J at 28 days, surpassing even the PP-reinforced mixtures. This sharp rise suggests a threshold effect, where higher fibre availability and better crack–fibre interaction lead to effective toughening despite the inherently brittle nature of basalt fibres. Notably, the CV at 0.25% BF was very low (1.15%), indicating consistent performance at this dosage.

The comparison between the 7-day and 28-day results also highlights the role of curing time. For all mixtures, toughness increased with age, reflecting the development of the matrix–fibre bond and the internal microstructure. The effect was more pronounced in BF-reinforced composites, possibly due to the improved mechanical anchorage in the stiffer cementitious matrix at later ages.

In general, the results clearly demonstrate the different toughening mechanisms associated with each type of fibre. PP fibres contribute to steady post-crack load transfer and ductility across all dosages, whereas BF fibres show significant toughness enhancement only at higher contents, where sufficient fibre bridging occurs. The combined analysis of the load–displacement response and toughness data confirms that the optimal fibre content and type must be carefully selected to balance strength, ductility, and energy absorption in the SCC mixtures.

## 4. Statistical Analysis

### 4.1. Statistical Analysis of Compressive Strength

These findings confirm that the mechanical performance of SCC is not governed solely by the dose of the fibres, but is also strongly influenced by the mechanical and geometric properties of the fibres. In particular, an optimum fibre content can be identified for each fibre type beyond which the strength tends to decrease.

A two-way analysis of variance (ANOVA) was conducted to assess the influence of fibre type (polypropylene, basalt, and none) and fibre volume content (0%, 0.025%, 0.05%, 0.075%, 0.125%, and 0.25%) on the 28-day compressive strength. Prior to ANOVA, the Shapiro–Wilk test was used to confirm the normality of the data (*p* > 0.05 in most cases), and Levene’s test confirmed homogeneity of variances (*p* = 0.157). The results, summarised in Table 5, indicate that both the fibre type and fibre content significantly affect the compressive strength at 28 days (*p* < 0.001). Moreover, a significant interaction effect (*p* < 0.001) was observed between fibre content and fibre type, indicating that the influence of fibre dosage varied depending on the fibre type.

These findings suggest that both the type and volume of fibres should be jointly considered when optimizing SCC mix designs for improved compressive strength.

The post hoc Tukey HSD test confirmed that almost all pairwise comparisons between SCC mixtures with varying fibre types and volume contents exhibited statistically significant differences in compressive strength at 28 days (*p* < 0.05). In particular, mixtures containing 0.05% and 0.075% PP fibres showed significantly higher compressive strengths than the reference mixture and other PP groups. Similarly, the SCC with 0.05% BF demonstrated a statistically higher strength than the reference SCC and the BF mixtures with 0.125% and 0.25%, which revealed the lowest strength values among all series. The statistical analysis also indicated that increasing the fibre volume beyond optimal values (approximately 0.05–0.075%) resulted in a significant decrease in compressive strength, particularly for basalt fibres. These results are consistent with the interaction effect observed in the two-way ANOVA and support the presence of an optimal fibre content range for maximizing compressive performance in SCC. The results of the pairwise comparisons are presented in the Supplementary Material, Table S5.

### 4.2. Statistical Analysis of Splitting Tensile Strength

To assess the effects of fibre type and volume fraction on the splitting tensile strength, a two-way analysis of variance (ANOVA) was performed using 28-day data. The independent variables included the fibre type (none, PP, BF) and fibre volume content (0%, 0.025%, 0.05%, 0.075%, 0.125%, and 0.25%). Prior to ANOVA, normality was verified using the Shapiro–Wilk test, and Levene’s test confirmed the homogeneity of variances (*p* > 0.05). The ANOVA results, summarised in Table 6, reveal that both the fibre type (*p* = 0.0054) and fibre volume content (*p* < 0.0001) had a statistically significant influence on the splitting tensile strength. Furthermore, the interaction effect between the fibre type and volume fraction was also statistically significant (*p* < 0.0001), indicating that the influence of fibre dosage depends strongly on the fibre type.

To further explore the pairwise differences between SCC mixtures, a post hoc Tukey HSD test was conducted. The results (provided in Supplementary Table S6) confirmed several statistically significant differences (*p* < 0.05) between the fibre dosages and types. Specifically, SCC mixtures containing 0.05% and 0.075% PP fibres exhibited a significantly higher tensile strength than the control mix and other PP series. Similarly, SCC-BF-0.05 showed significantly superior performance compared to both the reference and other BF mixes with higher fibre contents. Additionally, a notable reduction in tensile strength was observed between SCC-PP-0.125 and SCC-PP-0.25, confirming that fibre overdosing can lead to decreased performance due to impaired matrix cohesion and fibre agglomeration.

These statistical results align well with the trends observed in the experimental data (Table S2, Figure 3) and confirm that the optimal fibre dosages for improving the splitting tensile strength are approximately 0.05 to 0.125% for both fibre types. Fibre addition beyond this threshold may compromise matrix homogeneity and stress transfer efficiency, particularly in the case of basalt fibres due to their rigidity and hydrophilicity.

### 4.3. Statistical Analysis of Flexural Strength

A two-way analysis of variance (ANOVA) was conducted to evaluate the effects of fibre type and fibre volume fraction on the flexural strength of the SCC mixtures after 28 days of curing. Independent variables included fibre type (no, PP, BF) and fibre content (0%, 0.025%, 0.05%, 0.075%, 0.125%, and 0.25%). The assumptions of normality and homogeneity of variances were met, as confirmed by the Shapiro–Wilk and Levene’s tests (*p* > 0.05). The results, presented in Table 7, indicate that fibre content had a statistically significant effect on flexural strength (*p* < 0.0001), while fibre type alone was not a significant factor (*p* = 0.6201). However, the interaction between fibre type and fibre volume was statistically significant (*p* < 0.0001), indicating that the impact of fibre content on flexural strength depends on the type of fibre used.

A post hoc Tukey HSD test (see Supplementary Table S7) revealed several significant pairwise differences (*p* < 0.05). In particular, SCC mixtures containing 0.05% to 0.125% PP or BF fibres showed a significantly higher flexural strength than the reference SCC, with the highest values observed at 0.05% BF and 0.125% PP. A marked decline in flexural strength was found between SCC-PP-0.125 and SCC-PP-0.25, consistent with the hypothesis that an excessive fibre volume can reduce matrix homogeneity and adversely affect mechanical performance. These results confirm that while the type of fibre may not independently control flexural strength, its effectiveness is strongly influenced by dosage. The significant interaction effect highlights the importance of carefully selecting both fibre type and optimal volume fraction to maximise performance in SCC applications.

### 4.4. Statistical Analysis of Flexural Toughness

To investigate the effects of fibre type and content on the flexural toughness of SCC mixtures after 28 days, a two-way ANOVA was conducted. Fibre type (none, PP, BF) and fibre volume fraction (0%, 0.025%, 0.05%, 0.075%, 0.125%, and 0.25%) were selected as independent variables. The dependent variable was the measured flexural toughness, expressed in joules (J), calculated as the area under the load–displacement curve following the ASTM C1609 methodology [33].

The assumptions of normality and homoscedasticity were satisfied, allowing for valid use of ANOVA. The results, summarised in Table 8, indicate that all three factors (fibre type, fibre content, and their interaction) significantly affected flexural toughness (*p* < 0.0001 for all).

Post hoc comparisons using the Tukey HSD test (see Supplementary Table S8) revealed multiple statistically significant pairwise differences. In particular, PP-reinforced SCC showed substantial increases in flexural toughness at a moderate fibre content (0.075–0.125%), while the highest values were observed in BF-reinforced mixtures at a 0.25% content. This suggests that BF fibres, despite their brittleness, provide effective crack-bridging at higher dosages due to their high stiffness and energy absorption capacity. However, lower BF contents did not yield comparable improvements, underscoring the importance of reaching a threshold volume for effective fibre engagement.

The results also show that PP fibres are more effective in uniformly improving flexural toughness across a wider range of dosages, with lower variability compared to BF fibres at the same volume content. These findings reinforce the idea that both type and content must be optimised together to achieve a balanced toughness and post-crack performance in SCC.

It should be noted that statistical significance does not always equal practical significance in the design of structural concrete. Although ANOVA and Tukey tests revealed statistically significant differences between mixes, practical implications depend on the magnitude of improvement in mechanical and durability properties. For example, small but statistically significant variations in compressive strength may have limited structural impact, whereas large gains in post-cracking toughness and energy absorption (200–300%) are of clear practical importance for performance-based design. Effect sizes should therefore be considered alongside *p*-values when interpreting these findings.

## 5. Discussion

The comparative assessment of self-compacting concrete (SCC) reinforced with polypropylene (PP) and basalt (BF) fibres reveals notable distinctions in terms of mechanical performance, fracture behaviour, and practical applicability. Both types of fibres improved the post-cracking behaviour of the SCC, particularly in splitting tensile and flexural strength, but the mechanisms and degree of improvement varied depending on the stiffness of the fibre, the bonding characteristics, and the dispersion behaviour within the matrix.

Polypropylene fibres demonstrated a clear ability to enhance the ductility and fracture toughness of SCC at moderate contents (0.05–0.125% vol.), primarily by bridging microcracks and redistributing tensile stresses. The results indicated a consistent increase in both splitting tensile and flexural strength with increasing PP content up to an optimal dosage, beyond which a decline was observed. This trend was attributed to reduced workability and potential fibre clustering, which may lead to heterogeneous fibre dispersion and local weaknesses. These findings are in agreement with experimental studies by Chen et al. [34], who reported that PP fibres significantly improved fracture toughness and impact resistance in high-strength SCC, particularly at dosages below 0.2% vol. Similarly, Li et al. [35] observed an enhanced post-peak load-bearing capacity and improved crack distribution in PP-reinforced SCC due to fibre ductility and their capacity to restrain crack widening. Recent findings by Hussein and Ghalehnovi [36] confirmed that PP fibres, even at low dosages, effectively enhance the tensile strength and ductility of SCC by crack-bridging action. Furthermore, when combined with colloidal nanosilica, they improve the microstructure and durability of SCC by incorporating recycled aggregates, demonstrating a sustainable approach without compromising performance. Similarly, Katebi et al. [37] investigated SCC incorporating polypropylene fibres together with treated rubber powder and wash water, highlighting the potential for using waste materials in combination with fibres. Their results showed an improved tensile performance despite moderate reductions in compressive strength and workability, supporting the feasibility of such sustainable mix designs. In contrast, basalt fibres, due to their higher stiffness and better tensile strength, exhibited the superior enhancement of flexural and splitting tensile strength, especially at 28 days. The peak performance was observed at 0.125–0.25% vol., where the concrete displayed both higher peak loads and improved toughness indices. The higher relative increase in splitting tensile strength compared to flexural strength for the 0.05% BF mixture may be explained by the more uniform distribution and activation of fibres under splitting tensile loading, as well as the different stress states and crack propagation mechanisms involved in the two test methods. This is consistent with previous findings reported in the literature [38,39]. This was attributed to the strong interfacial bonding of basalt fibres to the cementitious matrix and their ability to efficiently transfer tensile loads across developing cracks. Prior research supports this, as noted by Al-Rousan et al. [40], who emphasised that basalt fibres provide consistent mechanical improvements and long-term durability without compromising thermal stability. Similarly, Akbulut and Guler [41] demonstrated that BF significantly improves the thermal stability and residual mechanical properties of self-compacting lightweight concrete after exposure to high temperatures, while slightly reducing workability. Additionally, Sathe and Kolte [42] reported that hybrid BF–PP systems offered a synergistic enhancement of energy absorption and post-cracking load capacity, outperforming mixes with single fibre types. An important distinction lies in the crack-control mechanisms. PP fibres, being flexible and hydrophobic, are more effective in controlling early-age shrinkage and microcracking, while BF fibres, being stiff and hydrophilic, enhance load transfer across macrocracks and resist propagation under sustained loading. This difference was reflected in the coefficient of variation (CV) values, where PP-reinforced mixes exhibited more stable behaviour at lower contents, whereas BF-reinforced mixes, particularly at higher dosages, displayed greater variability due to potential dispersion issues. These observations align with the results of Xue et al. [43] and Vedhasakthi and Chithra [44], who highlighted that the stiff nature of BF can contribute to uneven fibre distribution and microstructural stress concentrations when dosed excessively. In terms of fracture toughness, both fibres contributed significantly, with BF-reinforced SCC reaching higher energy absorption levels under flexural loading. Liang et al. [45] confirmed that fracture energy increased almost linearly with BF content up to 0.25%, while Fu et al. [46] proposed a flexural toughness model incorporating the hybrid action of PP and BF fibres, demonstrating improved accuracy in predicting post-crack performance. Our findings align with these results, showing a 5-fold increase in flexural toughness at 28 days compared to the reference mix for the optimal BF dosage. Despite these benefits, fibre addition had a detrimental effect on fresh SCC workability. Both fibre types decreased the flowability of the mix, with a more pronounced impact observed for BF at dosages ≥0.125%. This is consistent with studies by Ashteyat et al. [47] and Deng et al. [48], who found that an increased fibre content correlates with reduced slump flow and higher segregation resistance. In practical applications, this necessitates the optimisation of the admixture dosage and mixing procedure to ensure uniform fibre dispersion and adequate self-compacting ability.

From a design perspective, the results suggest that PP fibres are preferable when targeting shrinkage control and early-age tensile performance, whereas BF fibres are more suitable for enhancing flexural capacity and long-term fracture resistance. The combination of both types of fibre (hybridisation) has been shown to produce balanced mechanical benefits, as evidenced in the work of Chia et al. [49] and Zhang et al. [50], where hybrid SCC exhibited improved crack distribution and toughness under static and dynamic loads. Although hybrid systems were not investigated in this study, the complementary nature of PP and BF fibres provides a promising avenue for future research and structural applications, particularly in precast, pavement and high-durability elements. Importantly, this study did not include microstructural investigations. However, the observed macroscopic trends and statistical consistency between specimens are sufficient to validate mechanical findings. In general, the positive impact of both PP and BF fibres on fracture performance, energy absorption, and tensile resistance, combined with their availability and compatibility with SCC technology, supports their practical implementation in performance-based concrete design.

## 6. Conclusions

This study has demonstrated the effectiveness of polypropylene (PP) and basalt (BF) fibres in enhancing the mechanical performance and fracture toughness of self-compacting concrete (SCC). Based on the experimental results and the statistical analysis, the following conclusions can be drawn.

- Both PP and BF fibres significantly improved the splitting tensile strength, flexural strength, and flexural toughness of SCC, with the extent of improvement depending on the type of fibre and dosage.
- PP fibres proved to be more effective in the early ages (7 days), particularly at 0.05–0.125% volume, by controlling microcrack development and improving tensile stress distribution. The peak tensile strength increase reached 45% compared to the control mix.
- BF fibres offered superior post-cracking performance and toughness, especially at 28 days. Optimal performance was observed at 0.125–0.25% volume, where toughness increased by over 500% relative to the plain SCC.
- Excessive fibre content (≥0.25%) led to reduced workability and greater variability in results, indicating the need for dosage optimisation.
- Statistical evaluation using ANOVA and Tukey’s test confirmed significant differences between mixtures and validated observed trends.
- For practical design, PP fibres are suitable for improving early-age cracking resistance, while BF fibres are recommended for applications that require enhanced post-crack load-bearing capacity and energy absorption.
- The findings suggest that the hybridisation of PP and BF fibres could offer complementary benefits, and further studies on long-term durability, rheological effects, and hybrid systems are encouraged.

These results support the integration of low-volume, non-metallic fibres into the SCC mix design to improve performance in a sustainable and cost-effective manner.

## Figures and Tables

**Table 1:** Chemical constituents of cement and GGBS (in %).

Compound	Cement	GGBS
SiO_2_	20.19	33.14
Al_2_O_3_	4.30	13.55
Fe_2_O_3_	3.25	1.30
CaO	64.61	43.36
MgO	1.41	6.48
SO_3_	2.96	0.29
K_2_O	2.59	0.31
Na_2_O	0.26	0.29
Cl	0.111	0.006
LOI	3.41	0.76
Insoluble matter	0.48	0.31

**Table 2:** Properties of fibres.

Property	Polypropylene Fibres	Basalt Fibres
Density [kg/m^3^]	910	2700
Length [mm]	12	12
Diameter [μm]	25	13
Tensile strength [MPa]	305	1700
Elastic modulus [GPa]	35	70
Elongation at break [%]	10	2.4

**Table 3:** Mix proportions of self-compacting concrete mixtures.

Mix ID	Cement [kg/m^3^]	GGBFS [kg/m^3^]	Fine Aggregate [kg/m^3^]	Coarse Aggregate [kg/m^3^]	Water [kg/m^3^]	SUPERPLASTICIZER [% Binder]	Fibres Type	Fibre Content [% Vol.]
SCC-REF	350	300	980	400	210	3	None	0
SCC-PP-0.025	350	300	980	400	210	3	Polypropylene	0.025
SCC-PP-0.05	350	300	980	400	210	3	Polypropylene	0.05
SCC-PP-0.075	350	300	980	400	210	3	Polypropylene	0.075
SCC-PP-0.125	350	300	980	400	210	3	Polypropylene	0.125
SCC-PP-0.25	350	300	980	400	210	3	Polypropylene	0.25
SCC-BF-0.025	350	300	980	400	210	3	Basalt	0.025
SCC-BF-0.05	350	300	980	400	210	3	Basalt	0.05
SCC-BF-0.075	350	300	980	400	210	3	Basalt	0.075
SCC-BF-0.125	350	300	980	400	210	3	Basalt	0.125
SCC-BF-0.25	350	300	980	400	210	3	Basalt	0.25

**Table 4:** Fresh properties of self-compacting concretes.

Mix ID	Fibre Type	Fibre Content [% Vol.]	Slump Flow [mm]	T_500_ [s]	L-Box, PA [—]
SCC-REF	None	0	722.5	4.1	0.94
SCC-PP-0.025	Polypropylene	0.025	715	4.3	0.90
SCC-PP-0.05	Polypropylene	0.05	705.5	4.5	0.88
SCC-PP-0.075	Polypropylene	0.075	580.5	4.8	0.85
SCC-PP-0.125	Polypropylene	0.125	570	5.0	0.82
SCC-PP-0.25	Polypropylene	0.25	485	—	0.63
SCC-BF-0.025	Basalt	0.025	680	4.4	0.89
SCC-BF-0.05	Basalt	0.05	613.5	5.0	0.87
SCC-BF-0.075	Basalt	0.075	575	5.5	0.84
SCC-BF-0.125	Basalt	0.125	552	5.9	0.81
SCC-BF-0.25	Basalt	0.25	478.5	—	0.61

Note: The SCC-PP-0.25 and SCC-BF-0.25 mixtures did not satisfy the slump flow, the T_500_ flow time, and the PA criterion for SCC. However, their hardened properties were evaluated to allow for a comprehensive comparison and to demonstrate the effects of excessive fibre content on both workability and mechanical performance. “—” indicates that the mix did not reach a 500 mm spread; therefore, T_500_ could not be measured.

**Table 5:** Summary of results of two-way ANOVA for the compressive strength of 28 days of SCC mixtures. Effect of fibre type and fibre volume content on compressive strength (*p* < 0.05 considered statistically significant).

Source	Sum Sq	df	F	*p*-Value
Fibre type	1218.59	2	580.96	<0.001
Fibre content (vol. %)	4933.93	5	940.89	<0.001
Interaction (type × content)	586.63	10	55.93	<0.001
Residual	41.95	40	—	—

Note: “—” indicates that F and p-values are not applicable for the residual term in the ANOVA table.

**Table 6:** Two-way ANOVA results for splitting tensile strength of SCC after 28 days.

Source	Sum Sq	df	F	*p*-Value
Fibre type	0.834	2	8.66	0.0054
Fibre content (vol. %)	34.003	5	141.22	<0.0001
Interaction (type × content)	11.774	10	24.39	<0.0001
Residual	1.926	40	—	—

Note: “—” indicates that F and *p*-values are not applicable for the residual term in the ANOVA table.

**Table 7:** Two-way ANOVA results of flexural strength of SCC after 28 days.

Source	Sum Sq	df	F	*p*-Value
Fibre type	0.102	2	0.253	0.3201
Fibre content (vol. %)	55.989	5	55.633	<0.0001
Interaction (type × content)	22.772	10	211.314	<0.0001
Residual	4.428	22	—	—

Note: “—” indicates that F and p-values are not applicable for the residual term in the ANOVA table.

**Table 8:** Two-way ANOVA results for flexural toughness of SCC after 28 days.

Source	Sum Sq	df	F	*p*-Value
Fibre type	150.06	2	156.11	<0.0001
Fibre content (vol. %)	1209.39	5	503.25	<0.0001
Interaction (type × content)	361.17	10	75.15	<0.0001
Residual	12.02	25	—	—

Note: “—” indicates that F and p-values are not applicable for the residual term in the ANOVA table.

## Data Availability

The original contributions presented in this study are included in the article/Supplementary Material. Further inquiries can be directed to the corresponding authors.

## Supplementary Materials

The following supporting information can be downloaded at: https://www.mdpi.com/article/10.3390/ma18163833/s1, Table S1: Compressive strength of SCC mixtures at 7 and 28 days; Table S2: Splitting tensile strength of SCC mixtures at 7 and 28 days; Table S3: Flexural strength of SCC mixtures at 7 and 28 days; Table S4: Flexural toughness of SCC mixtures at 7 and 28 days; Table S5: Post hoc Tukey’s HSD test results for 28-day compressive strength of SCC mixtures. Pairwise comparison of fibre-reinforced SCC mixtures; Table S6: Post hoc Tukey’s HSD test results for 28-day splitting tensile strength of SCC mixtures. Pairwise comparisons using the Tukey HSD test for splitting tensile strength of SCC mixtures after 28 days. Statistically significant differences (*p* < 0.05) are indicated in the ‘reject’ column; Table S7: Tukey HSD Test–Flexural Strength (28 days). Pairwise comparisons using the Tukey HSD test for flexural strength of SCC mixtures after 28 days. Statistically significant differences (*p* < 0.05) are indicated in the ‘reject’ column; Table S8: Tukey HSD Test – Flexural Toughness (28 days). Pairwise comparisons using the Tukey HSD test for flexural toughness of SCC mixtures after 28 days. Statistically significant differences (*p* < 0.05) are indicated in the ‘reject’ column.
