# Supplementary material for: Comparative Study on Mechanical Performance and Toughness of High-Performance Self-Compacting Concrete with Polypropylene and Basalt Fibres
Source: Materials (Basel). 2025 Aug 15;18(16):3833. doi: 10.3390/ma18163833 (PMC12387742; doi:10.3390/ma18163833)
Supplement: Supplementary file 1 [file materials-18-03833-s001.zip › materials-3782082-supplementary.pdf]

Table S1. Compressive strength of SCC mixtures at 7 and 28 days.

Mix ID	Fibre type	Fibre content [% vol.]	7 days [MPa]	SD [MPa]	CV [%]	28 days [MPa]	SD [MPa]	CV [%]
SCC-REF	None	0	36.6	0.85	2.33	68.2	0.43	0.64
SCC-PP-0.025	Polypropylene	0.025	39.9	0.32	0.81	71.9	0.13	0.19
SCC-PP-0.05	Polypropylene	0.05	47.9	0.80	1.67	73.8	1.16	1.57
SCC-PP-0.075	Polypropylene	0.075	41.5	0.69	1.68	72.9	0.22	0.30
SCC-PP-0.125	Polypropylene	0.125	40.8	0.30	0.74	68.8	0.27	0.39
SCC-PP-0.25	Polypropylene	0.25	38.4	0.55	1.43	66.3	0.76	1.14
SCC-BF-0.025	Basalt	0.025	37.3	0.66	1.77	69.7	1.02	1.46
SCC-BF-0.05	Basalt	0.05	45.6	1.69	3.71	70.9	1.36	1.93
SCC-BF-0.075	Basalt	0.075	40.5	0.86	2.13	69.4	1.44	2.08
SCC-BF-0.125	Basalt	0.125	33.8	0.99	2.93	62.3	2.50	4.01
SCC-BF-0.25	Basalt	0.25	27.3	0.58	2.14	57.5	2.04	3.55

Table S2. Splitting tensile strength of SCC mixtures at 7 and 28 days.

Mix ID	Fibre type	Fibre content [% vol.]	7 days [MPa]	SD [MPa]	CV [%]	28 days [MPa]	SD [MPa]	CV [%]
SCC-REF	None	0	3.07	0.08	2.57	4.63	0.02	0.53
SCC-PP-0.025	Polypropylene	0.025	3.42	0.02	0.48	5.34	0.03	0.49
SCC-PP-0.05	Polypropylene	0.05	3.83	0.07	1.73	6.04	0.06	0.96
SCC-PP-0.075	Polypropylene	0.075	3.96	0.07	1.72	6.26	0.09	1.41
SCC-PP-0.125	Polypropylene	0.125	4.25	0.07	1.64	6.70	0.16	2.39
SCC-PP-0.25	Polypropylene	0.25	3.22	0.11	3.44	5.60	0.46	8.32
SCC-BF-0.025	Basalt	0.025	3.45	0.09	2.52	5.52	0.47	8.51
SCC-BF-0.05	Basalt	0.05	3.75	0.05	1.36	6.75	0.18	2.61
SCC-BF-0.075	Basalt	0.075	3.59	0.04	1.12	5.92	0.24	4.11
SCC-BF-0.125	Basalt	0.125	3.32	0.06	1.78	5.43	0.08	1.45
SCC-BF-0.25	Basalt	0.25	3.17	0.05	1.63	4.78	0.09	1.97

Table S3. Flexural strength of SCC mixtures at 7 and 28 days.

Mix ID	Fibre type	Fibre content [% vol.]	7 days [MPa]	SD [MPa]	CV [%]	28 days [MPa]	SD [MPa]	CV [%]
SCC-REF	None	0	3.02	0.08	2.62	6.41	0.23	3.55
SCC-PP-0.025	Polypropylene	0.025	3.41	0.03	0.82	7.00	0.11	1.52
SCC-PP-0.05	Polypropylene	0.05	3.86	0.13	3.40	7.55	0.10	1.31
SCC-PP-0.075	Polypropylene	0.075	4.02	0.05	1.32	8.22	0.44	5.36
SCC-PP-0.125	Polypropylene	0.125	4.35	0.24	5.43	9.08	1.22	13.44
SCC-PP-0.25	Polypropylene	0.25	3.51	0.13	3.62	7.16	0.53	7.42
SCC-BF-0.025	Basalt	0.025	4.87	0.19	3.95	8.56	0.10	1.17
SCC-BF-0.05	Basalt	0.05	5.50	0.06	1.13	8.83	0.21	2.40
SCC-BF-0.075	Basalt	0.075	5.18	0.18	3.51	8.01	0.33	4.08
SCC-BF-0.125	Basalt	0.125	4.05	0.20	5.05	7.25	0.11	1.52
SCC-BF-0.25	Basalt	0.25	3.51	0.13	3.62	6.85	0.03	0.38

Table S4. Flexural toughness of SCC mixtures at 7 and 28 days.

Mix ID	Fibre type	Fibre content [% vol.]	7 days [J]	SD [J]	CV [%]	28 days [J]	SD [J]	CV [%]
SCC-REF	None	0	2.292	0.600	26.19	2.628	0.526	20.01
SCC-PP-0.025	Polypropylene	0.025	3.587	0.180	5.01	4.208	0.119	2.84
SCC-PP-0.05	Polypropylene	0.05	4.299	0.816	18.99	4.747	0.377	7.94
SCC-PP-0.075	Polypropylene	0.075	9.937	0.106	1.07	10.834	0.890	8.21
SCC-PP-0.125	Polypropylene	0.125	10.166	0.131	1.29	13.063	1.653	12.65
SCC-PP-0.25	Polypropylene	0.25	12.523	1.283	10.25	13.153	0.179	1.36
SCC-BF-0.025	Basalt	0.025	2.448	0.316	12.91	2.808	0.396	14.11
SCC-BF-0.05	Basalt	0.05	2.559	0.355	13.89	3.211	0.429	13.35
SCC-BF-0.075	Basalt	0.075	2.640	0.331	12.55	3.616	0.210	5.81

SCC-BF-0.125	Basalt	0.125	3.489	0.337	9.67	5.482	1.089	19.87
SCC-BF-0.25	Basalt	0.25	11.990	0.789	6.58	15.702	0.180	1.15

Table S5. Post hoc Tukey's HSD test results for 28-day compressive strength of SCC mixtures. Pairwise comparison of fibre-reinforced SCC mixtures.

	group1	group2	meandiff	p-adj
2	BF-0.025	BF-0.125	-7.4127	0
3	BF-0.025	BF-0.25	-12.2367	0
5	BF-0.025	PP-0.05	4.1133	0.0001
6	BF-0.025	PP-0.075	3.21	0.0031
8	BF-0.025	PP-0.25	-3.4367	0.0012
11	BF-0.05	BF-0.125	-8.548	0
12	BF-0.05	BF-0.25	-13.372	0
14	BF-0.05	PP-0.05	2.978	0.0077
17	BF-0.05	PP-0.25	-4.572	0
18	BF-0.05	REF-0.0	-2.6913	0.0229
19	BF-0.075	BF-0.125	-7.0447	0
20	BF-0.075	BF-0.25	-11.8687	0
21	BF-0.075	PP-0.025	2.5947	0.0324
22	BF-0.075	PP-0.05	4.4813	0
23	BF-0.075	PP-0.075	3.578	0.0007
25	BF-0.075	PP-0.25	-3.0687	0.0054
27	BF-0.125	BF-0.25	-4.824	0
28	BF-0.125	PP-0.025	9.6393	0
29	BF-0.125	PP-0.05	11.526	0
30	BF-0.125	PP-0.075	10.6227	0
31	BF-0.125	PP-0.125	6.526	0
32	BF-0.125	PP-0.25	3.976	0.0001
33	BF-0.125	REF-0.0	5.8567	0
34	BF-0.25	PP-0.025	14.4633	0
35	BF-0.25	PP-0.05	16.35	0
36	BF-0.25	PP-0.075	15.4467	0
37	BF-0.25	PP-0.125	11.35	0
38	BF-0.25	PP-0.25	8.8	0
39	BF-0.25	REF-0.0	10.6807	0
42	PP-0.025	PP-0.125	-3.1133	0.0002
43	PP-0.025	PP-0.25	-5.6633	0
44	PP-0.025	REF-0.0	-3.7827	0
46	PP-0.05	PP-0.125	-5	0
47	PP-0.05	PP-0.25	-7.55	0
48	PP-0.05	REF-0.0	-5.6693	0
49	PP-0.075	PP-0.125	-4.0967	0
50	PP-0.075	PP-0.25	-6.6467	0
51	PP-0.075	REF-0.0	-4.766	0
52	PP-0.125	PP-0.25	-2.55	0.0043

Table S6. Post hoc Tukey's HSD test results for 28-day splitting tensile strength of SCC mixtures. Pairwise comparisons using the Tukey HSD test for splitting tensile strength of SCC mixtures after 28 days. Statistically significant differences ($p < 0.05$) are indicated in the 'reject' column.

group1	group2	meandiff	p-adj	lower	upper	reject
BF-0.025	BF-0.05	1.2287	0.0	0.6175	1.8398	True
PP-0.125	PP-0.25	-1.104	0.0	-1.5361	-0.6719	True
PP-0.075	REF-0.0	-1.63	0.0	-2.0621	-1.1979	True
PP-0.05	REF-0.0	-1.4113	0.0	-1.8435	-0.9792	True
PP-0.025	PP-0.125	1.3617	0.0	0.9295	1.7938	True
PP-0.025	PP-0.075	0.92	0.0	0.4879	1.3521	True
BF-0.25	PP-0.125	1.9187	0.0	1.3894	2.4479	True
BF-0.25	PP-0.075	1.477	0.0	0.9477	2.0063	True
BF-0.25	PP-0.05	1.2583	0.0	0.7291	1.7876	True
BF-0.125	PP-0.125	1.2703	0.0	0.7411	1.7996	True
PP-0.125	REF-0.0	-2.0717	0.0	-2.5038	-1.6395	True
BF-0.075	REF-0.0	-1.29	0.0	-1.8193	-0.7607	True
BF-0.075	BF-0.25	-1.137	0.0	-1.7481	-0.5259	True
BF-0.05	REF-0.0	-2.1243	0.0	-2.6536	-1.5951	True
BF-0.05	PP-0.25	-1.1567	0.0	-1.6859	-0.6274	True
PP-0.25	REF-0.0	-0.9677	0.0	-1.3998	-0.5355	True
BF-0.025	PP-0.125	1.176	0.0	0.6467	1.7053	True
BF-0.025	REF-0.0	-0.8957	0.0	-1.4249	-0.3664	True
BF-0.05	PP-0.025	-1.4143	0.0	-1.9436	-0.8851	True
BF-0.05	BF-0.125	-1.323	0.0	-1.9341	-0.7119	True
BF-0.05	BF-0.25	-1.9713	0.0	-2.5825	-1.3602	True
PP-0.025	REF-0.0	-0.71	0.0001	-1.1421	-0.2779	True
PP-0.025	PP-0.05	0.7013	0.0001	0.2692	1.1335	True
BF-0.125	PP-0.075	0.8287	0.0002	0.2994	1.3579	True
PP-0.075	PP-0.25	-0.6623	0.0003	-1.0945	-0.2302	True
BF-0.25	PP-0.25	0.8147	0.0003	0.2854	1.3439	True
PP-0.05	PP-0.125	0.6603	0.0003	0.2282	1.0925	True
BF-0.125	REF-0.0	-0.8013	0.0003	-1.3306	-0.2721	True
BF-0.075	PP-0.125	0.7817	0.0005	0.2524	1.3109	True
BF-0.025	PP-0.075	0.7343	0.0012	0.2051	1.2636	True
BF-0.05	BF-0.075	-0.8343	0.0016	-1.4455	-0.2232	True
BF-0.05	PP-0.05	-0.713	0.0019	-1.2423	-0.1837	True
BF-0.025	BF-0.25	-0.7427	0.007	-1.3538	-0.1315	True
BF-0.125	PP-0.05	0.61	0.0128	0.0807	1.1393	True
BF-0.075	PP-0.025	-0.58	0.0216	-1.1093	-0.0507	True
BF-0.125	BF-0.25	-0.6483	0.0296	-1.2595	-0.0372	True
BF-0.25	PP-0.025	0.557	0.0319	0.0277	1.0863	True
PP-0.05	PP-0.25	-0.4437	0.0399	-0.8758	-0.0115	True
PP-0.075	PP-0.125	0.4417	0.0415	0.0095	0.8738	True
BF-0.025	PP-0.05	0.5157	0.0619	-0.0136	1.0449	False
BF-0.05	PP-0.075	-0.4943	0.0855	-1.0236	0.0349	False
BF-0.075	BF-0.125	-0.4887	0.2241	-1.0998	0.1225	False
BF-0.025	BF-0.075	0.3943	0.5174	-0.2168	1.0055	False
BF-0.075	PP-0.075	0.34	0.5238	-0.1893	0.8693	False
BF-0.075	PP-0.25	-0.3223	0.5991	-0.8516	0.2069	False
PP-0.025	PP-0.25	0.2577	0.6278	-0.1745	0.6898	False
PP-0.05	PP-0.075	0.2187	0.8135	-0.2135	0.6508	False
BF-0.025	PP-0.025	-0.1857	0.9793	-0.7149	0.3436	False
BF-0.125	PP-0.25	0.1663	0.9907	-0.3629	0.6956	False
BF-0.25	REF-0.0	-0.153	0.9951	-0.6823	0.3763	False
BF-0.075	PP-0.05	0.1213	0.9993	-0.4079	0.6506	False
BF-0.125	PP-0.025	-0.0913	0.9999	-0.6206	0.4379	False
BF-0.05	PP-0.125	-0.0527	1.0	-0.5819	0.4766	False
BF-0.025	BF-0.125	-0.0943	1.0	-0.7055	0.5168	False
BF-0.025	PP-0.25	0.072	1.0	-0.4573	0.6013	False

Table S7. Tukey HSD Test – Flexural Strength (28 days). Pairwise comparisons using the Tukey HSD test for flexural strength of SCC mixtures after 28 days. Statistically significant differences ($p < 0.05$) are indicated in the 'reject' column.

group1	group2	meandiff	p-adj	lower	upper	reject
PP-0.125	REF-0.0	-2.6777	0.0	-3.9872	-1.3682	True
BF-0.05	REF-0.0	-2.4243	0.0001	-3.7338	-1.1148	True
BF-0.25	PP-0.125	2.2353	0.0002	0.9258	3.5448	True
BF-0.025	REF-0.0	-2.1473	0.0003	-3.4568	-0.8378	True
PP-0.025	PP-0.125	2.0867	0.0004	0.7772	3.3962	True
BF-0.05	BF-0.25	-1.982	0.0008	-3.2915	-0.6725	True
PP-0.125	PP-0.25	-1.9253	0.0011	-3.2348	-0.6158	True
BF-0.125	PP-0.125	1.8393	0.002	0.5298	3.1488	True
BF-0.05	PP-0.025	-1.8333	0.002	-3.1428	-0.5238	True
PP-0.075	REF-0.0	-1.8133	0.0023	-3.1228	-0.5038	True
BF-0.025	BF-0.25	-1.705	0.0046	-3.0145	-0.3955	True
BF-0.05	PP-0.25	-1.672	0.0056	-2.9815	-0.3625	True
BF-0.075	REF-0.0	-1.6033	0.0086	-2.9128	-0.2938	True
BF-0.05	BF-0.125	-1.586	0.0096	-2.8955	-0.2765	True
BF-0.025	PP-0.025	-1.5563	0.0115	-2.8658	-0.2468	True
PP-0.05	PP-0.125	1.536	0.013	0.2265	2.8455	True
BF-0.025	PP-0.25	-1.395	0.0304	-2.7045	-0.0855	True
BF-0.25	PP-0.075	1.371	0.035	0.0615	2.6805	True
BF-0.025	BF-0.125	-1.309	0.0501	-2.6185	0.0005	False
BF-0.05	PP-0.05	-1.2827	0.0582	-2.5922	0.0268	False
PP-0.025	PP-0.075	1.2223	0.0814	-0.0872	2.5318	False
BF-0.075	BF-0.25	-1.161	0.1131	-2.4705	0.1485	False
PP-0.05	REF-0.0	-1.1417	0.1251	-2.4512	0.1678	False
BF-0.075	PP-0.125	1.0743	0.1754	-0.2352	2.3838	False
PP-0.075	PP-0.25	-1.061	0.1871	-2.3705	0.2485	False
BF-0.075	PP-0.025	-1.0123	0.2351	-2.3218	0.2972	False
BF-0.025	PP-0.05	-1.0057	0.2423	-2.3152	0.3038	False
BF-0.125	PP-0.075	0.975	0.2776	-0.3345	2.2845	False
PP-0.075	PP-0.125	0.8643	0.432	-0.4452	2.1738	False
BF-0.075	PP-0.25	-0.851	0.4531	-2.1605	0.4585	False
BF-0.125	REF-0.0	-0.8383	0.4734	-2.1478	0.4712	False
BF-0.05	BF-0.075	-0.821	0.5019	-2.1305	0.4885	False
BF-0.075	BF-0.125	-0.765	0.5961	-2.0745	0.5445	False
PP-0.25	REF-0.0	-0.7523	0.6176	-2.0618	0.5572	False
BF-0.25	PP-0.05	0.6993	0.7059	-0.6102	2.0088	False
PP-0.05	PP-0.075	0.6717	0.7496	-0.6378	1.9812	False
BF-0.05	PP-0.075	-0.611	0.8358	-1.9205	0.6985	False
PP-0.025	REF-0.0	-0.591	0.8605	-1.9005	0.7185	False
PP-0.025	PP-0.05	0.5507	0.9037	-0.7588	1.8602	False
BF-0.025	BF-0.075	-0.544	0.9099	-1.8535	0.7655	False
BF-0.025	PP-0.125	0.5303	0.9219	-0.7792	1.8398	False
BF-0.075	PP-0.05	-0.4617	0.9665	-1.7712	0.8478	False
BF-0.25	REF-0.0	-0.4423	0.9748	-1.7518	0.8672	False
BF-0.125	BF-0.25	-0.396	0.9884	-1.7055	0.9135	False
PP-0.05	PP-0.25	-0.3893	0.9897	-1.6988	0.9202	False
BF-0.025	PP-0.075	-0.334	0.9968	-1.6435	0.9755	False
BF-0.25	PP-0.25	0.31	0.9983	-0.9995	1.6195	False
BF-0.125	PP-0.05	0.3033	0.9985	-1.0062	1.6128	False
BF-0.025	BF-0.05	0.277	0.9993	-1.0325	1.5865	False
BF-0.125	PP-0.025	-0.2473	0.9997	-1.5568	1.0622	False
BF-0.05	PP-0.125	0.2533	0.9997	-1.0562	1.5628	False
BF-0.075	PP-0.075	0.21	0.9999	-1.0995	1.5195	False
BF-0.25	PP-0.025	0.1487	1.0	-1.1608	1.4582	False

BF-0.125	PP-0.25	-0.086	1.0	-1.3955	1.2235	False
PP-0.025	PP-0.25	0.1613	1.0	-1.1482	1.4708	False

Table S8. Tukey HSD Test – Flexural Toughness (28 days). Pairwise comparisons using the Tukey HSD test for flexural toughness of SCC mixtures after 28 days. Statistically significant differences ($p < 0.05$) are indicated in the 'reject' column.

group1	group2	meandiff	p-adj	lower	upper	reject
BF-0.125	BF-0.25	10.2193	0.0	8.2209	12.2178	True
BF-0.075	PP-0.125	9.4477	0.0	7.4492	11.4461	True
BF-0.075	PP-0.25	9.537	0.0	7.5386	11.5354	True
PP-0.125	REF-0.0	-10.4352	0.0	-12.1659	-8.7045	True
BF-0.125	PP-0.075	5.3517	0.0	3.3532	7.3501	True
BF-0.125	PP-0.125	7.581	0.0	5.5826	9.5794	True
BF-0.125	PP-0.25	7.6703	0.0	5.6719	9.6688	True
BF-0.25	PP-0.025	-11.4937	0.0	-13.4921	-9.4952	True
BF-0.25	PP-0.05	-10.955	0.0	-12.9534	-8.9566	True
BF-0.25	PP-0.075	-4.8677	0.0	-6.8661	-2.8692	True
BF-0.25	REF-0.0	-13.0735	0.0	-14.8042	-11.3428	True
PP-0.025	PP-0.075	6.626	0.0	4.6276	8.6244	True
PP-0.025	PP-0.125	8.8553	0.0	6.8569	10.8538	True
PP-0.025	PP-0.25	8.9447	0.0	6.9462	10.9431	True
PP-0.05	PP-0.075	6.0873	0.0	4.0889	8.0858	True
PP-0.05	PP-0.125	8.3167	0.0	6.3182	10.3151	True
PP-0.05	PP-0.25	8.406	0.0	6.4076	10.4044	True
PP-0.075	REF-0.0	-8.2058	0.0	-9.9365	-6.4751	True
BF-0.075	PP-0.075	7.2183	0.0	5.2199	9.2168	True
BF-0.075	BF-0.25	12.086	0.0	10.0876	14.0844	True
PP-0.25	REF-0.0	-10.5245	0.0	-12.2552	-8.7938	True
BF-0.025	BF-0.25	12.8937	0.0	10.8952	14.8921	True
BF-0.025	PP-0.075	8.026	0.0	6.0276	10.0244	True
BF-0.05	PP-0.25	9.9413	0.0	7.9429	11.9398	True
BF-0.05	PP-0.125	9.852	0.0	7.8536	11.8504	True
BF-0.025	PP-0.125	10.2553	0.0	8.2569	12.2538	True
BF-0.05	PP-0.075	7.6227	0.0	5.6242	9.6211	True
BF-0.025	PP-0.25	10.3447	0.0	8.3462	12.3431	True
BF-0.05	BF-0.25	12.4903	0.0	10.4919	14.4888	True
BF-0.125	REF-0.0	-2.8542	0.0002	-4.5849	-1.1235	True
BF-0.025	BF-0.125	2.6743	0.003	0.6759	4.6728	True
BF-0.25	PP-0.125	-2.6383	0.0035	-4.6368	-0.6399	True
BF-0.25	PP-0.25	-2.549	0.0052	-4.5474	-0.5506	True
PP-0.05	REF-0.0	-2.1185	0.008	-3.8492	-0.3878	True
PP-0.075	PP-0.25	2.3187	0.0138	0.3202	4.3171	True
BF-0.05	BF-0.125	2.271	0.0168	0.2726	4.2694	True
PP-0.075	PP-0.125	2.2293	0.0199	0.2309	4.2278	True
BF-0.025	PP-0.05	1.9387	0.0628	-0.0598	3.9371	False
BF-0.075	BF-0.125	1.8667	0.0821	-0.1318	3.8651	False
PP-0.025	REF-0.0	-1.5798	0.0958	-3.3105	0.1509	False
BF-0.05	PP-0.05	1.5353	0.2494	-0.4631	3.5338	False
BF-0.025	PP-0.025	1.4	0.3643	-0.5984	3.3984	False
BF-0.125	PP-0.025	-1.2743	0.493	-3.2728	0.7241	False
BF-0.075	REF-0.0	-0.9875	0.6416	-2.7182	0.7432	False
BF-0.075	PP-0.05	1.131	0.6518	-0.8674	3.1294	False
BF-0.05	PP-0.025	0.9967	0.7908	-1.0018	2.9951	False
BF-0.025	BF-0.075	0.8077	0.9297	-1.1908	2.8061	False
BF-0.125	PP-0.05	-0.7357	0.9603	-2.7341	1.2628	False
BF-0.05	REF-0.0	-0.5832	0.978	-2.3139	1.1475	False

BF-0.075	PP-0.025	0.5923	0.9912	-1.4061	2.5908	False
PP-0.025	PP-0.05	0.5387	0.9958	-1.4598	2.5371	False
BF-0.05	BF-0.075	0.4043	0.9996	-1.5941	2.4028	False
BF-0.025	BF-0.05	0.4033	0.9996	-1.5951	2.4018	False
BF-0.025	REF-0.0	-0.1798	1.0	-1.9105	1.5509	False
PP-0.125	PP-0.25	0.0893	1.0	-1.9091	2.0878	False
